# CAF-Driven Mechanotransduction via Collagen Remodeling Accelerates Tumor Cell Cycle Progression

**DOI:** 10.3390/gels11080642

**Published:** 2025-08-13

**Authors:** Yating Xiao, Yingying Jiang, Ting Bao, Xin Hu, Xiang Wang, Xiaoning Han, Linhong Deng

**Affiliations:** 1 Institute of Biomedical Engineering and Health Sciences, Changzhou University, Changzhou 213164, China; xyt1021514035@163.com (Y.X.); wangxiang@cczu.edu.cn (X.W.); 2 School of Pharmacy, Changzhou University, Changzhou 213164, China; 3 School of Medical and Health Engineering, Changzhou University, Changzhou 213164, China

**Keywords:** collagen hydrogels, cancer-associated fibroblasts, mechanotransduction, matrix stiffening, YAP/TAZ, G1/S transition

## Abstract

Cancer-associated fibroblasts (CAFs) restructure collagen hydrogels via actomyosin-driven fibril bundling and crosslinking, increasing polymer density to generate mechanical stress that accelerates tumor proliferation. Conventional hydrogel models lack spatial heterogeneity, thus obscuring how localized stiffness gradients regulate cell cycle progression. To address this, we developed a collagen hydrogel-based microtissue platform integrated with programmable microstrings (single/double tethering), enabling real-time quantification of gel densification mechanics and force transmission efficiency. Using this system combined with FUCCI cell cycle biosensors and molecular perturbations, we demonstrate that CAF-polarized contraction increases hydrogel stiffness (350 → 775 Pa) and reduces pore diameter (5.0 → 1.9 μm), activating YAP/TAZ nuclear translocation via collagen–integrin–actomyosin cascades. This drives a 2.4-fold proliferation increase and accelerates G1/S transition in breast cancer cells. Pharmacological inhibition of YAP (verteporfin), actomyosin (blebbistatin), or collagen disruption (collagenase) reversed mechanotransduction and proliferation. Partial rescue upon CYR61 knockdown revealed compensatory effector networks. Our work establishes CAF-remodeled hydrogels as biomechanical regulators of tumor growth and positions gel-based mechanotherapeutics as promising anti-cancer strategies.

## 1. Introduction

The extracellular matrix (ECM) serves as a biomechanical scaffold that governs tumor proliferation and cell cycle progression through dynamic mechanochemical crosstalk. Cancer-associated fibroblasts (CAFs) [1] dynamically remodel collagen hydrogel architecture through actomyosin-driven bundling and crosslinking enhancement, progressively increasing polymer density to generate mechanical stress [2,3]. This hydrogel restructuring directly activates integrin–FAK signaling and actomyosin-mediated contraction, generating mechanical stress that directly activates integrin–FAK signaling and YAP/TAZ transcriptional programs [4,5]. These mechanotransduction cascades bypass canonical growth factor pathways to accelerate G1/S phase transition and sustain mitotic activity, thereby promoting chemoresistance and metastatic dissemination [6]. Critically, CAF-generated forces—including polarized traction and compressive stress—reshape ECM architecture into spatially heterogeneous stiffness gradients (e.g., aligned collagen fibers, solid stress hotspots), which further amplify YAP/TAZ nuclear translocation and cyclin-dependent kinase activation [7,8]. This biomechanical reprogramming establishes a permissive niche for unchecked tumor growth, positioning CAF-ECM mechanics as a central regulator of oncogenic cell cycle dynamics.

Despite established roles of mechanical cues in tumor progression, current experimental models fail to recapitulate the dynamic spatial heterogeneity of force gradients within tumor microenvironments. Conventional collagen hydrogels with fixed crosslinking densities fail to capture CAF-mediated time-dependent stiffening and anisotropic force transmission [9,10]. Although tunable stiffness gels quantify bulk properties, their inability to spatially control boundary constraints impedes modeling of localized stress gradients in fibrillar networks—critical drivers of anisotropic ECM stiffening and collagen realignment at invasive fronts. While advanced platforms (e.g., tunable stiffness gels) quantify bulk mechanical properties, they lack spatial control over boundary constraints, rendering them incapable of modeling localized force transmission via integrin-bound fibrils or cell–cell junctions [11,12]. This limitation obscures three fundamental gaps: (i) How polarized CAF contraction generates spatially resolved stiffness gradients to accelerate tumor proliferation; (ii) whether collagen–integrin–YAP mechanotransduction directly couples force magnitude to G1/S checkpoint bypass; and (iii) to what extent functional redundancy among YAP effectors sustains proliferation under mechanical stress. Critically, the absence of technologies for real-time mapping of force-dependent cell cycle dynamics—particularly under defined mechanical boundaries—impedes mechanistic dissection of stroma-driven tumor growth.

To address this, we developed a collagen hydrogel-based microtissue platform integrated with programmable microstrings [13,14]. This system uniquely quantifies spatiotemporal evolution of gel densification mechanics during fibroblast-driven remodeling, while concurrently mapping with programmable mechanical boundaries (single/double tethering). This system uniquely quantifies dynamic force transmission efficiency during fibroblast-driven matrix densification while concurrently mapping cell cycle progression via FUCCI biosensors and YAP mechanotransduction under spatial constraints. Subsequent analyses reveal force-dependent acceleration of G1/S transition through collagen–integrin–actomyosin–YAP cascades, with compensatory effector networks sustaining proliferation upon partial pathway inhibition.

In summary, this work delineates a mechanobiological axis wherein CAF-polarized contraction bypasses G1/S checkpoints via collagen–integrin–YAP signaling to accelerate tumor proliferation. Our dual-microstring platform pioneers real-time mapping of mechanical heterogeneity and cell cycle dynamics, offering a transformative tool for spatial mechanobiology studies. These findings position YAP/TAZ and stromal mechanics as actionable targets for anti-cancer mechanotherapeutics.

## 2. Results and Discussion

### 2.1. Model Establishment and Biomechanical Characterization

**Figure 1:**
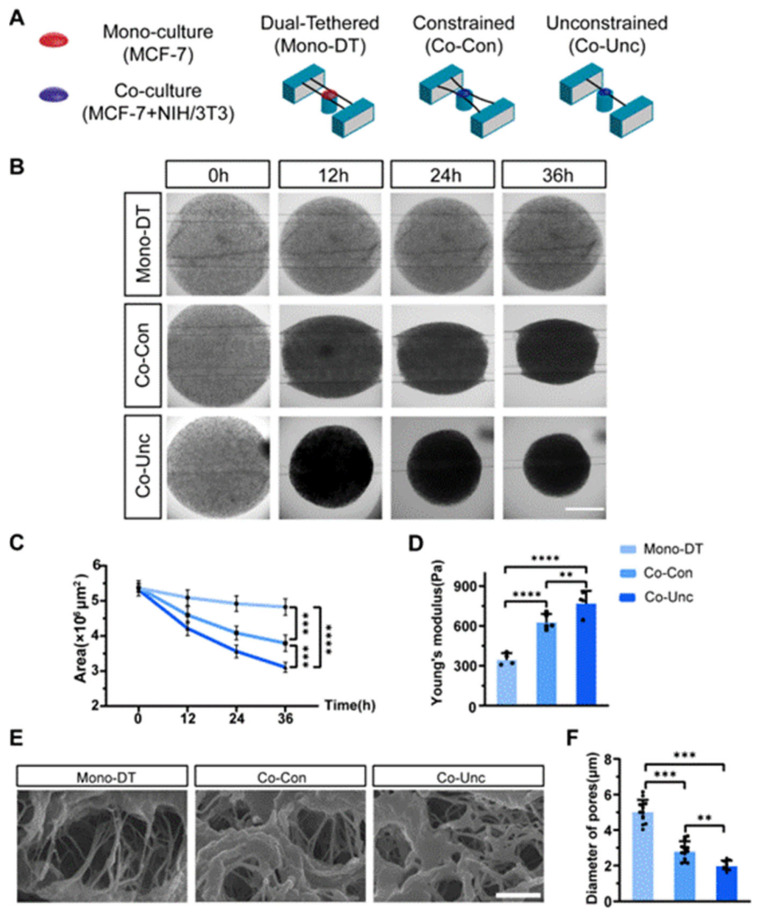
Construction of a tumor microtissue contraction model and its biomechanical properties characterization under different mechanical conditions. (**A**) Schematics of three mechanical configurations: Mono-DT (MCF-7 alone, dual-tethered), and Co-Con (MCF-7 + NIH/3T3, dual-tethered), Co-Unc (co-culture, single-tethered). PDMS microstrings (k = 0.08 μN/μm) impose programmable boundaries. (**B**) Bright-field images showing contraction dynamics over 36 h (scale bar = 1 mm). (**C**) Changes in planar area of tumor microtissues with different mechanical conditions. (**D**) Young’s modulus increased from 350 ± 35 Pa (Mono-DT) to 775 ± 45 Pa (Co-Unc) after 36 h. (**E**) SEM scans of the internal collagen structure of microtissues with different mechanical conditions. Scale bar = 2 μm. (**F**) Pore diameter reduction reflects matrix densification (** *p* < 0.01, *** *p* < 0.001, **** *p* < 0.0001).

We developed a 3D microtissue model incorporating PDMS-based microstring sensors (150 μm diameter, spring constants k = 0.08 μN/μm) to investigate mechanical interactions between stromal fibroblasts and tumor cells. Three experimental groups were established: (1) Mono-culture dual-tethered (Mono-DT) microtissues containing MCF-7 breast cancer cells alone in collagen, anchored between two microstrings; (2) co-culture constrained (Co-Con) microtissues with MCF-7 and NIH/3T3 fibroblasts co-embedded in collagen and tethered to dual microstrings to restrict contraction; and (3) co-culture unconstrained (Co-Unc) microtissues, where co-cultured cells were anchored to a single microstring to enable unrestricted radial contraction (Figure 1A). Time-lapse imaging over 36 h revealed distinct contraction dynamics: Mono-DT microtissues exhibited minimal contraction (projected area reduction: 9.4 ± 1.2%) with no detectable microstring deflection, whereas Co-Con microtissues condensed into spindle-shaped geometries (area reduction: 34.1 ± 2.6%), and Co-Unc microtissues contracted radially, achieving a 43.7 ± 3.3% reduction in projected area (Figure 1B,C). The enhanced contraction in Co-Unc microtissues likely stems from unopposed fibroblast traction forces, which polarize along the free boundary to maximize collagen fibril compaction, whereas dual-tethered configurations (Co-Con) distribute forces bidirectionally, limiting net densification. AFM-based mechanical characterization demonstrated significant differences in stiffness, with Mono-DT microtissues maintaining a Young’s modulus of 350 ± 35 Pa, while Co-Con and Co-Unc microtissues stiffened to 632 ± 48 Pa and 775 ± 45 Pa, respectively (Figure 1D). SEM imaging further confirmed fibroblast-driven collagen matrix remodeling, as average pore diameters decreased from 5.0 ± 1.9 μm in Mono-DT microtissues to 2.8 ± 1.3 μm in Co-Con and 1.9 ± 1.0 μm in Co-Unc groups (Figure 1E,F). These results collectively validate the successful establishment of a tunable microtissue model that recapitulates fibroblast-mediated mechanical stress and matrix densification under spatially defined boundary conditions, providing a physiologically relevant platform to study stromal–epithelial mechanobiology in tumor-like microenvironments.

### 2.2. Mechanical Regulation of Tumor Cell Proliferation

While stromal-derived mechanical forces are known to accelerate tumor progression by promoting cell cycle progression, the dynamic coupling between contraction magnitude and proliferation kinetics in spatially heterogeneous 3D microenvironments—particularly under defined mechanical boundaries—remains poorly characterized. This gap limits our understanding of how localized force gradients, such as those generated by polarized fibroblast traction, directly modulate mitotic entry and tumor growth.

**Figure 2:**
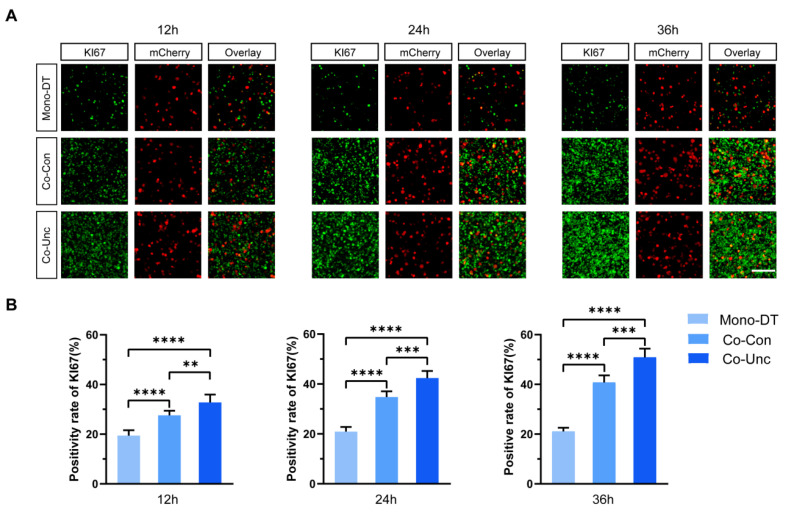
Analysis of KI67 proliferative activity of MCF-7 cells in tumor microtissues under different mechanical conditions. (**A**) Expression of KI67 proliferation markers (green) in MCF-7 tumor cells (red) within microtissues under different mechanical conditions at 12 h, 24 h and 36 h of culture, respectively. Scale bar = 150 μm. (**B**) Statistics of KI67 positivity rate of MCF-7 tumor cells in microtissues cultured under different mechanical conditions at 12 h, 24 h and 36 h, respectively. KI67+ cells (%) show force-dependent increase (mean ± SD; *n* = 6 fields/group; ** *p* < 0.01, *** *p* < 0.001, **** *p* < 0.0001 vs. Mono-DT). Co-Unc: 2.4-fold higher proliferation at 36 h.

To assess how mechanical boundary conditions influence tumor cell proliferation, MCF-7 cells were transfected with the pPAmCherry1-N1 plasmid to stably express mCherry fluorescent protein, enabling unambiguous identification and tracking within co-cultured microtissues containing unlabeled NIH/3T3 fibroblasts. KI67 expression (a marker of proliferative activity) and phosphorylated histone H3 (PH3, a mitotic marker) were quantified specifically in mCherry-positive MCF-7 cells across the three microtissue groups. In Mono-DT microtissues, KI67-positive cells remained consistently low (19.4 ± 1.8% at 12 h; 21.7 ± 2.9% at 36 h), with no significant temporal increase (Figure 2A,B). In contrast, Co-Con microtissues exhibited time-dependent proliferation enhancement, with KI67 positivity rising from 26.7 ± 3.2% at 12 h to 40.7 ± 4.1% at 36 h. Strikingly, Co-Unc microtissues displayed the most rapid proliferation, reaching 51.9 ± 2.7% KI67 positivity by 36 h, a 2.4-fold increase compared to Mono-DT (Figure 2B).

**Figure 3:**
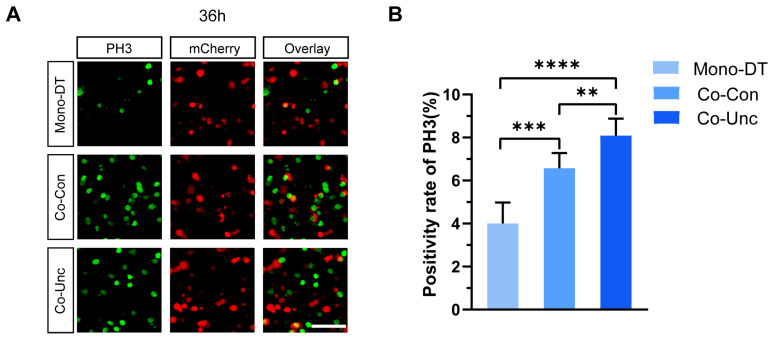
Mitotic entry is accelerated by mechanical stress. (**A**) Fluorescence expression of PH3 mitotic markers in MCF-7 tumor cells within microtissues under different mechanical conditions. Scale bar = 150 μm. (**B**) Statistics of PH3 positivity of MCF-7 tumor cells in microtissues with different mechanical conditions. PH3+ cells (%) in Co-Unc doubled vs. Mono-DT (** *p* < 0.01, *** *p* < 0.001, **** *p* < 0.0001). Data represent 3 independent experiments.

PH3 staining further corroborated these findings: mitotic indices in Co-Unc microtissues (8.1 ± 1.7%) were double those in Mono-DT (4.0 ± 0.8%) and significantly higher than in Co-Con (6.5 ± 1.5%) at 36 h (Figure 3A,B). These results demonstrate that fibroblast-generated mechanical forces drive tumor cell proliferation in a contraction-dependent manner, with unconstrained mechanical boundaries (Co-Unc) eliciting the strongest pro-proliferative effects. The correlation between contraction magnitude, matrix stiffening, and proliferation kinetics underscores the critical role of biomechanical cues in regulating tumor growth.

### 2.3. Mechanical Regulation of Tumor Cell Cycle Progression

**Figure 4:**
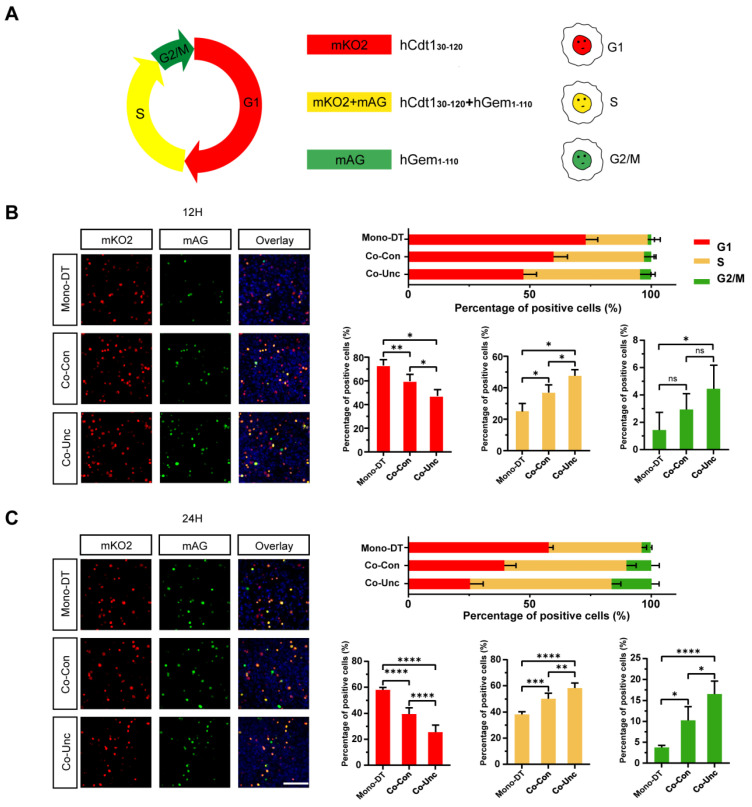
Unconstrained mechanical boundaries accelerate the G1/S transition. (**A**) Schematic diagram of the principle of the FUCCI cell cycle indicator system. (**B**) FUCCI fluorescence expression of MCF-7 tumor cells in microtissues under different mechanical conditions at 12 h of culture (left) and cell cycle distribution statistics (right). Scale bar = 150 μm. (**C**) FUCCI fluorescence expression of MCF-7 tumor cells in microtissues with different mechanical conditions at 24 h of culture (left) and cell cycle distribution statistics (right). Cell cycle distribution at 12/24 h post-thymidine release. Co-Unc microtissues show 44.6% S-phase entry at 12 h vs. 19.0% in Mono-DT (* *p* < 0.05, ** *p* < 0.01, *** *p* < 0.001, **** *p* < 0.0001). Scale bar = 150 μm. *n* = 200+ cells/group from 3 replicates.

To delineate the impact of mechanical forces on cell cycle dynamics, MCF-7 cells were transfected with a fluorescent ubiquitination-based cell cycle indicator (FUCCI) system, enabling live tracking of cell cycle phases through distinct fluorescence signals: red (G1 phase), yellow (G1/S transition and S phase), and green (G2/M phase) (Figure 4A). Following thymidine-mediated synchronization to arrest cells at the G1/S boundary, microtissues were released into contraction-permissive conditions, and cell cycle progression was monitored over 36 h.

In Mono-DT microtissues, cells exhibited delayed cell cycle entry, with only 19.0 ± 2.7% transitioning into S phase by 12 h and 45.1 ± 5.9% remaining in G1 phase at 24 h. In contrast, Co-Con microtissues displayed accelerated progression, with 30.8 ± 4.3% of cells entering S phase by 12 h and 56.6 ± 7.4% advancing to G2/M phase by 24 h. The most pronounced effect was observed in Co-Unc microtissues, where 44.6 ± 5.2% of cells entered S phase within 12 h, and 58.3 ± 6.9% progressed to G2/M phase by 24 h, demonstrating a near-complete bypass of G1 checkpoint constraints (Figure 4B,C) (Supplementary Figure S1).

By 36 h, Co-Unc microtissues exhibited a marked reduction in G1-phase cells (55.8 ± 6.3%) compared to Mono-DT (84.3 ± 3.8%), alongside a higher proportion of cells re-entering subsequent cycles. These results indicate that fibroblast-generated mechanical forces expedite G1/S transition and shorten overall cycle duration, facilitating rapid tumor cell proliferation under unconstrained mechanical conditions.

Mechanical stress imposed by stromal fibroblasts disrupts cell cycle checkpoint regulation, preferentially accelerating G1/S phase transition and promoting continuous cycling. This mechano-dependent acceleration underscores the role of biomechanical cues in sustaining tumor growth through enhanced mitotic activity.

### 2.4. Mechanistic Insights into Mechanotransduction-Driven Proliferation

To dissect the molecular mechanisms by which fibroblast-generated mechanical forces drive tumor cell proliferation, we systematically interrogated the roles of mechanosensitive transcriptional regulators, cytoskeletal dynamics, and extracellular matrix (ECM) integrity. The experimental approach was designed to validate a hierarchical signaling cascade linking mechanical stress to cell cycle acceleration, with emphasis on spatial and temporal regulation.

#### 2.4.1. YAP/TAZ Activation as a Central Mechanotransduction Hub

**Figure 5:**
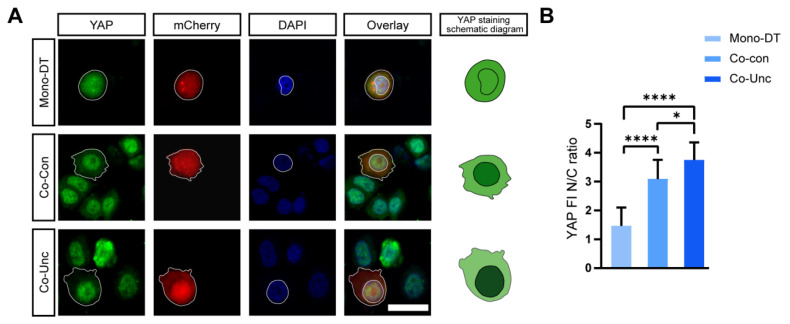
YAP nuclear translocation precedes G1/S acceleration. (**A**) Immunofluorescence staining map of YAP/TAZ nuclear localization (left) and its pattern (right). Scale bar = 30 μm. (**B**) Quantitative analysis of YAP/TAZ nuclear/plasmic fluorescence intensity ratio (* *p* < 0.05, **** *p* < 0.0001).

YAP/TAZ are established mediators of mechanical signaling, yet their activation dynamics in heterotypic 3D microenvironments remain poorly defined. To test whether fibroblast-induced forces directly activate YAP/TAZ in tumor cells, we performed immunofluorescence analysis of nuclear YAP localization. In Co-Unc microtissues, nuclear-to-cytoplasmic YAP intensity ratios (3.8 ± 0.6) were 2.7-fold higher than in Mono-DT (1.4 ± 0.2), confirming force-dependent nuclear translocation (Figure 5A). Pharmacological inhibition of YAP/TAZ with verteporfin (10 μM) reduced nuclear ratios to 1.7 ± 0.3 and suppressed KI67 positivity from 51.9 ± 2.7% to 22.2 ± 3.4% in Co-Unc microtissues, directly linking YAP/TAZ activity to proliferation (Figure 5B). These results establish YAP/TAZ as indispensable transducers of mechanical stress, converting stromal-derived forces into pro-proliferative transcriptional programs.

#### 2.4.2. Actomyosin Contractility as a Mechanical Signal Transducer

**Figure 6:**
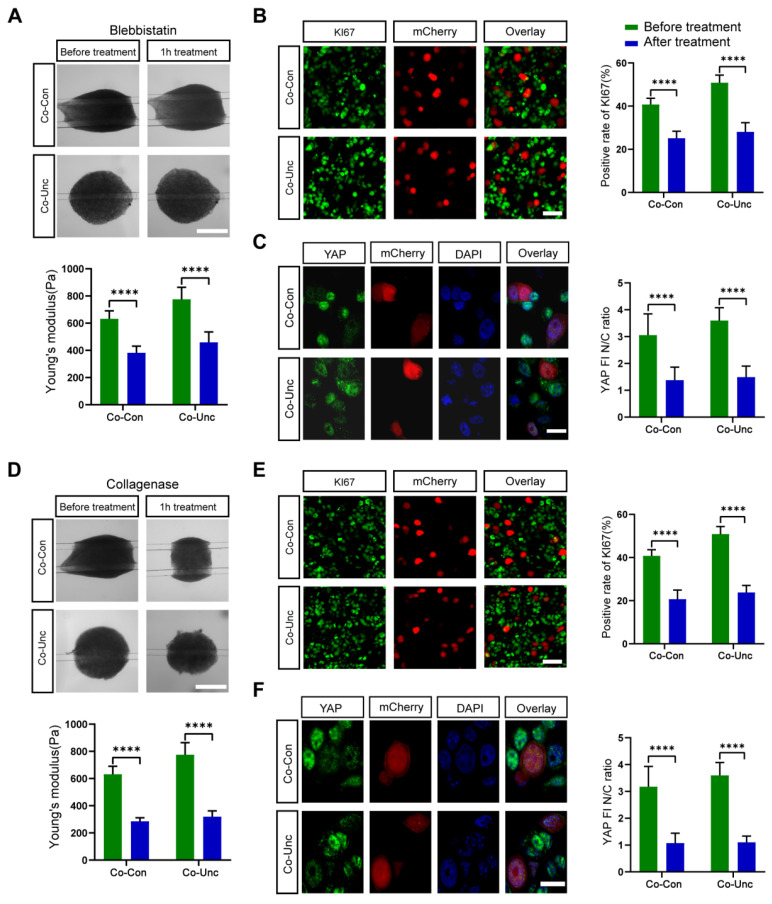
Regulatory effects of drug interventions on mechanical properties, YAP/TAZ signaling and tumor proliferation in co-cultured microtissues. (**A**) Representative time-lapse images of co-cultured microtissues after Blebbistatin (5 μm) treatment (top) and their Young’s modulus changes (bottom). Scale bar = 1 mm. (**B**) KI67 immunostaining images of MCF-7 in co-cultured microtissues after Blebbistatin treatment (left) and its KI67 positivity statistics (right panel). Scale bar = 50 μm. (**C**) Immunofluorescence staining images of YAP/TAZ nuclear localization of MCF-7 in co-cultured microtissues after Blebbistatin treatment (left) and its nuclear/plasmic fluorescence intensity ratio (right). Scale bar = 30 μm. (**D**) Representative time-lapse images of collagenase-treated (0.5 mg/mL) co-cultured microtissues (top) and their Young’s modulus changes (bottom). Scale bar = 1 mm. (**E**) KI67 immunostaining images (left) of MCF-7 in co-cultured microtissues after collagenase treatment and its KI67 positivity statistics (right)) (**** *p* < 0.0001). Scale bar = 50 μm. (**F**) Immunofluorescence staining images of YAP/TAZ nuclear localization of MCF-7 in co-cultured microtissues after collagenase treatment (left) and its nuclear/plasmic fluorescence intensity ratio (right). Scale bar = 30 μm.

Actomyosin-generated tension is hypothesized to propagate mechanical signals from fibroblasts to tumor cells. To validate this, we inhibited myosin II ATPase activity with blebbistatin (5 μM), aiming to decouple force generation from downstream signaling. Blebbistatin treatment reduced microtissue stiffness by 41% (Co-Unc: 775 ± 45 Pa → 458 ± 33 Pa) and abolished YAP nuclear localization (nuclear ratio: 1.4 ± 0.5 vs. 3.8 ± 0.6 in controls). Proliferation rates (KI67: 28.0 ± 4.5%) reverted to near-Mono-DT levels (Figure 6A–C). Actomyosin contractility is essential for both force transmission and YAP activation, confirming that tumor cells sense stromal forces via cytoskeletal remodeling. Fibroblast-generated tension likely propagates via integrin-bound collagen fibrils or direct cell–cell junctions, inducing actomyosin reorganization in MCF-7 cells to activate YAP/TAZ.

#### 2.4.3. Collagen Matrix Integrity Enables Mechanosignaling

**Figure 7:**
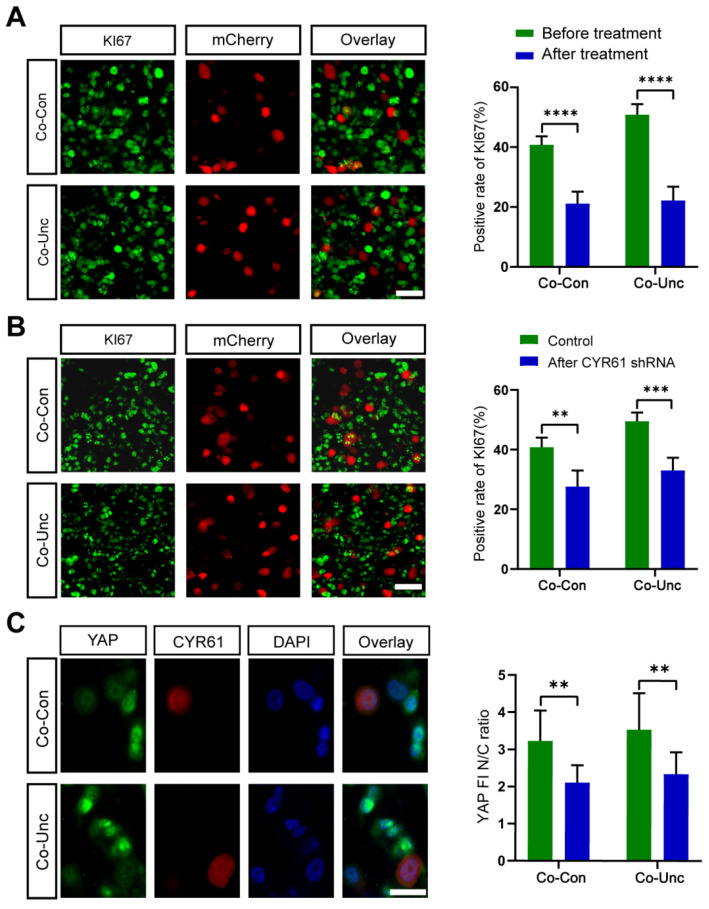
Regulation of mechanically driven tumor proliferation by CYR61, a downstream target gene of the YAP/TAZ signaling pathway. (**A**) Representative immunofluorescence staining images of KI67 of MCF-7 in co-cultured microtissues treated with Verteporfin (10 μM) for 36 h (left) and its KI67 positivity statistics (right). Scale bar = 50 μm. (**B**) Representative immunofluorescence staining images of KI67 of MCF-7 in co-culture microtissues with shRNA knockdown of CYR61 (left) and its KI67 positivity statistics (right). Scale bar = 50 μm. (**C**) Images of YAP/TAZ immunofluorescence staining of MCF-7 in co-cultured microtissues after CYR61 knockdown (left) and its nuclear/plasmic fluorescence intensity ratio (right)) (** *p* < 0.01, *** *p* < 0.001, **** *p* < 0.0001). Scale bar = 30 μm.

Collagen networks transmit mechanical signals through integrin adhesions. To assess whether ECM integrity is required for YAP activation, we disrupted collagen fibers with collagenase (0.5 mg/mL). Collagenase treatment reduced stiffness to 319 ± 24 Pa (Co-Unc) and diminished YAP nuclear ratios (1.1 ± 0.2) and KI67 positivity (23.8 ± 3.5%), mimicking Mono-DT phenotypes (Figure 7A). Intact collagen matrices are critical for force transmission, as matrix disruption uncouples integrin–ECM interactions and deactivates YAP/TAZ, highlighting the interdependence of ECM structure and mechanotransduction.

#### 2.4.4. Functional Redundancy of YAP/TAZ Downstream Effectors

To evaluate therapeutic potential, we targeted YAP-regulated genes CYR61, which are implicated in tumor proliferation. SiRNA-mediated knockdown of CYR61 reduced KI67 positivity in Co-Unc microtissues (51.9 ± 2.7% → 33.1 ± 4.3%) but failed to fully reverse cell cycle acceleration. Residual proliferation suggests compensatory activation of alternative YAP targets (e.g., ANKRD1) or stromal-derived paracrine signals (Figure 7B,C). Interpretation: Functional redundancy among YAP effectors underscores the robustness of mechano-proliferative networks, necessitating multi-target strategies to disrupt tumor growth.

Fibroblast-generated mechanical forces activate YAP/TAZ through actomyosin contractility and collagen-dependent integrin signaling, bypassing cell cycle checkpoints to accelerate tumor proliferation. While CYR61 are key effectors, their partial contribution reveals compensatory mechanisms that sustain proliferation under mechanical stress. These findings delineate a mechanobiological axis wherein stromal forces, cytoskeletal tension, and ECM remodeling converge on YAP/TAZ to drive tumor progression, offering actionable targets for disrupting biomechanical feedforward loops.

### 2.5. Discussion

This study elucidates the mechanobiological cascade by which fibroblast-generated mechanical forces drive tumor cell proliferation through YAP/TAZ-mediated cell cycle acceleration in a 3D co-culture microtissue model. By integrating tunable mechanical boundaries, live-cell cycle tracking, and molecular pathway inhibition, we demonstrate that stromal-derived contractility enhances ECM stiffening, activates YAP/TAZ nuclear translocation, and accelerates G1/S transition in breast cancer cells. Critically, collagen hydrogel stiffening acts as biomechanical memory, sustaining YAP/TAZ activation post-force removal. This mirrors stress-relaxation behaviors observed in fibrillar gels [2,15,16], where residual matrix tension persists after cellular contraction ceases.

This study demonstrates that fibroblast-mediated matrix stiffening in unconstrained co-culture microtissues (Co-Unc) robustly activates YAP/TAZ nuclear translocation, thereby accelerating G1/S transition and tumor proliferation. These findings align with the established role of YAP/TAZ as integrators of mechanical signals, where cytoskeletal tension and ECM stiffness synergistically regulate their activity [17]. Notably, Dasgupta and McCollum [18] previously highlighted that actomyosin contractility governs YAP/TAZ nuclear localization upon Hippo pathway inactivation, a dependency directly validated here through blebbistatin-induced myosin II inhibition. Importantly, our work extends these insights to a 3D heterotypic microenvironment, revealing the critical role of spatial mechanical heterogeneity. In Co-Unc microtissues, polarized fibroblast contraction generated localized stiffness gradients, as evidenced by SEM showing collagen pore reduction to 1.9 μm. This spatial heterogeneity likely mimics the mechanical landscape at invasive tumor fronts, where YAP activation peaks due to unopposed stromal forces [9,19]. Such context-dependent mechanoresponsiveness underscores the limitations of traditional 2D models in recapitulating tumor edge dynamics.

We demonstrate that CAF contractility reprograms collagen hydrogel topology through fibril bundling and crosslinking enhancement (Figure 1E,F), elevating Young’s modulus from 350 Pa to 775 Pa. This restructuring creates anisotropic permeability gradients (pore diameter reduction: 5.0 to 1.9 μm) that amplify force transmission—a process absent in static hydrogels with homogeneous polymer networks. Such CAF-driven gel densification establishes micromechanical hotspots that spatially coordinate YAP activation, mimicking stiffness-encoded signaling niches at invasive tumor fronts.

While CYR61 knockdown partially suppressed proliferation (51.9% to 33.1%), residual activity implies compensatory mechanisms involving alternative YAP targets (e.g., ANKRD1) or stromal paracrine factors. Foster et al. identified cross-regulation between MRTF-SRF and YAP-TEAD pathways, supporting functional redundancy among mechanosensitive effectors [20]. Additionally, Ishihara et al. revealed that CAF-secreted prosaposin enhances cancer cell survival independently of YAP/TAZ, suggesting multimodal stromal–tumor crosstalk [21]. A limitation of this study is the lack of systematic screening for all YAP-regulated genes or paracrine mediators. Future single-cell transcriptomic analyses [22] could comprehensively map redundant signaling networks. Notably, Barbazan et al. recently showed that CAF-generated compressive forces directly modulate cancer cell YAP localization, implying that combined targeting of CAF contractility (e.g., ROCK or Src inhibition) and YAP transcriptional activity may enhance therapeutic efficacy [23].

The mechanistic insights from this study offer actionable strategies for targeting biomechanical signaling in cancer therapy. Pharmacological inhibition of YAP/TAZ, such as verteporfin, could disrupt mechanotransduction-driven proliferation, as demonstrated by its efficacy in reverting nuclear YAP ratios to baseline levels. Similarly, interventions against stromal mechanical forces—via collagen crosslink breakers like ALT-711 or actomyosin inhibitors such as blebbistatin—may attenuate matrix stiffening and YAP activation, as supported by Jang et al. and Calvo et al. [24,25] Furthermore, the 3D microtissue platform developed here provides a physiologically relevant model for high-throughput drug screening, particularly for compounds targeting mechanosensitive pathways. Recent advances in tumor-on-chip systems and multi-organ platforms underscore the potential of integrating such models into preclinical pipelines to evaluate combinatorial therapies [26], such as pairing YAP inhibitors with immune checkpoint blockade, which may synergistically disrupt stromal–tumor mechanical crosstalk [27].

While this study elucidates the CAF-stiffness-YAP/TAZ axis, several limitations warrant attention. First, the reliance on a single cancer cell line (MCF-7) limits generalizability; incorporating patient-derived organoids or heterogeneous co-cultures could enhance translational relevance. Future studies will validate these mechanisms in patient-derived organoids to capture clinically relevant heterogeneity. Second, the exclusion of ERK/MAPK and Wnt pathways, known mechanotransduction regulators [17,28], leaves their interplay with YAP/TAZ unresolved. Multi-omics profiling could map cross-regulatory networks and identify compensatory pathways. Third, spatial heterogeneity in mechanical signaling remains underexplored. Techniques such as atomic force microscopy-based stiffness mapping [9] or spatial transcriptomics [22] could resolve force-dependent gene expression gradients. Addressing these gaps will refine our understanding of tumor mechanopathology and accelerate the development of mechanotherapeutics.

## 3. Conclusions

In summary, this work delineates a mechanobiological axis wherein stromal forces, cytoskeletal tension, and ECM remodeling converge on YAP/TAZ to drive tumor proliferation. By bypassing G1/S checkpoints and sustaining mitotic activity, biomechanical cues create a permissive niche for cancer growth. Targeting actomyosin contractility, collagen crosslinking, or YAP/TAZ activity may disrupt this feedforward loop, offering new avenues for combating biomechanically driven malignancies. Our 3D microtissue platform, with its ability to recapitulate spatially heterogeneous mechanical niches, provides a versatile tool for future explorations of tumor–stroma crosstalk and mechanotherapeutic development.

## 4. Materials and Methods

### 4.1. Silicon Mold Fabrication

Microstring silicon molds were fabricated using Deep Reactive Ion Etching (DRIE; Suzhou Wenhao, Suzhou, China). The design comprised two parallel grooves (50 μm height × 2 mm width × 4 mm length) interconnected by four microstrings (15 mm length × 200 μm width, spaced 1 mm apart). Etching parameters included 200 sccm SF_6_, 100 W RF power, and 30 mTorr chamber pressure at 20 °C. Molds were plasma-cleaned (O_2_, 100 W, 5 min) before PDMS replication.

### 4.2. PDMS Microstring Preparation

Sylgard 184 (Dow Corning, Midland, MI, USA, Cat# 1673921) was mixed 10:1 (*w*/*w*) base curing agent, degassed, and cast into molds. After curing (80 °C, 4 h), microstrings were anchored between PDMS standers (5 × 5 × 10 mm). Spring constants (k = 0.08 ± 0.005 μN/μm) were calibrated via AFM indentation (JPK NanoWizard 3), with linear elasticity confirmed (R^2^ > 0.99, 0–100 μm deflection range).

### 4.3. Cell Culture and Transfection

MCF-7 (ATCC HTB-22) and NIH/3T3 (ATCC, Manassas, VA, USA CRL-1658) cells were cultured in DMEM (Gibco, Grand Island, NY, USA, Cat # 11965092) supplemented with 10% FBS (Gibco, Grand Island, NY, USA, Cat# 16000044) and 1% penicillin–streptomycin (Gibco, Grand Island, NY, USA, Cat# 15140122). For live imaging, MCF-7 cells were transfected with pBOB-EF1-FastFUCCI-Puro (Addgene, Cambridge, MA, USA, # 86849) or pPAmCherry1-N1 (Addgene, Cambridge, MA, USA, # 31928) using Lipofectamine 3000 (Thermo, Waltham, MA, USA, Cat# L3000001), achieving > 80% efficiency.

### 4.4. Microtissue Model Construction

Three models were prepared: Mono-DT: 1 × 10^6^ MCF-7 cells in 8 μL collagen I (4 mg/mL, Advanced Biomatrix Cat# 5005) tethered between two microstrings.Co-Con: MCF-7 + NIH/3T3 (1:1 ratio, 1 × 10^6^ total cells) in collagen, dual-tethered. Co-Unc: Identical co-culture single-tethered to one microstring. Neutralized collagen (9:1 collagen:0.1 M NaOH) was mixed with cells, pipetted onto microstrings, polymerized (37 °C, 40 min), and submerged in 2 mL of medium.

### 4.5. Structural and Mechanical Characterization

Microtissues were fixed (3% glutaraldehyde, 4 °C, 24 h), critical-point dried, and imaged via Zeiss Sigma 300 SEM (5 kV). Pore diameters were measured from 10 random SEM fields using ImageJ (v1.53, National Institutes of Health, Bethesda, MD, USA). Young’s modulus was quantified by AFM (Bruker MLCT-O10 cantilever; 20 μm bead tip) with 1 μm/s indentation over 4 × 4 grids (50 × 50 μm area), analyzed via the Hertz model (JPKSPM v6.3). Contraction force was calculated from microstring deflection: F = k × (d_0_ − d_t_), where d_0_ = 700 μm.

### 4.6. Cell Cycle and Signaling Analysis

MCF-7 cells were synchronized at G1/S via thymidine double-block (2.5 mM, 17 h treatment → 10 h release → 17 h re-treatment; >90% efficiency). For proliferation, microtissues were stained with KI67 (Abcam, Cambridge, UK, ab15580, 1:500) or Phospho-Histone H3 (CST, Danvers, MA, USA, 3377, 1:500) and secondary AF488 antibody (Thermo, Waltham, MA, USA, A-11008, 1:500). FUCCI cell-cycle phases were quantified using ImageJ ROI Manager (National Institutes of Health, Bethesda, MD, USA). YAP nucleocytoplasmic ratios were determined from immunofluorescence (CST, Danvers, MA, USA, 14074, 1:200) relative to DAPI/MCherry masks. CYR61 was knocked down using shRNA (RiboBio Sanming, Fujian, China; 5′-GGACGAAAGUGUUCCAACA-3′; >70% efficiency). Drug treatments (Verteporfin 10 μM, blebbistatin 5 μM, and collagenase 0.5 mg/mL) were applied at 36 h for 1 h.

### 4.7. Data Processing and Statistics

Images were analyzed in ImageJ v1.53: ROIs were generated via thresholding and “Fit Spline” smoothing. Proliferation rates = (KI67+ or PH3+ cells)/DAPI+ cells. Statistical significance was determined by one-way ANOVA + Tukey post hoc test (GraphPad Prism v9), with data as mean ± SD (*n* ≥ 3). *p* < 0.05 was considered significant.

## Data Availability

The datasets generated during and/or analyzed during the current study are available from the corresponding author on reasonable request.

## Supplementary Materials

The following supporting information can be downloaded at https://www.mdpi.com/article/10.3390/gels11080642/s1, Figure S1: Analysis of cell cycle dynamics of MCF-7 cells in tumor microtissues under different mechanical conditions.
