# Supplementary material for: CAF-Driven Mechanotransduction via Collagen Remodeling Accelerates Tumor Cell Cycle Progression
Source: Gels. 2025 Aug 13;11(8):642. doi: 10.3390/gels11080642 (PMC12385777; doi:10.3390/gels11080642)
Supplement: Supplementary file 1 [file gels-11-00642-s001.zip › gels-3769210-supplementary.pdf]

Supplementary Material

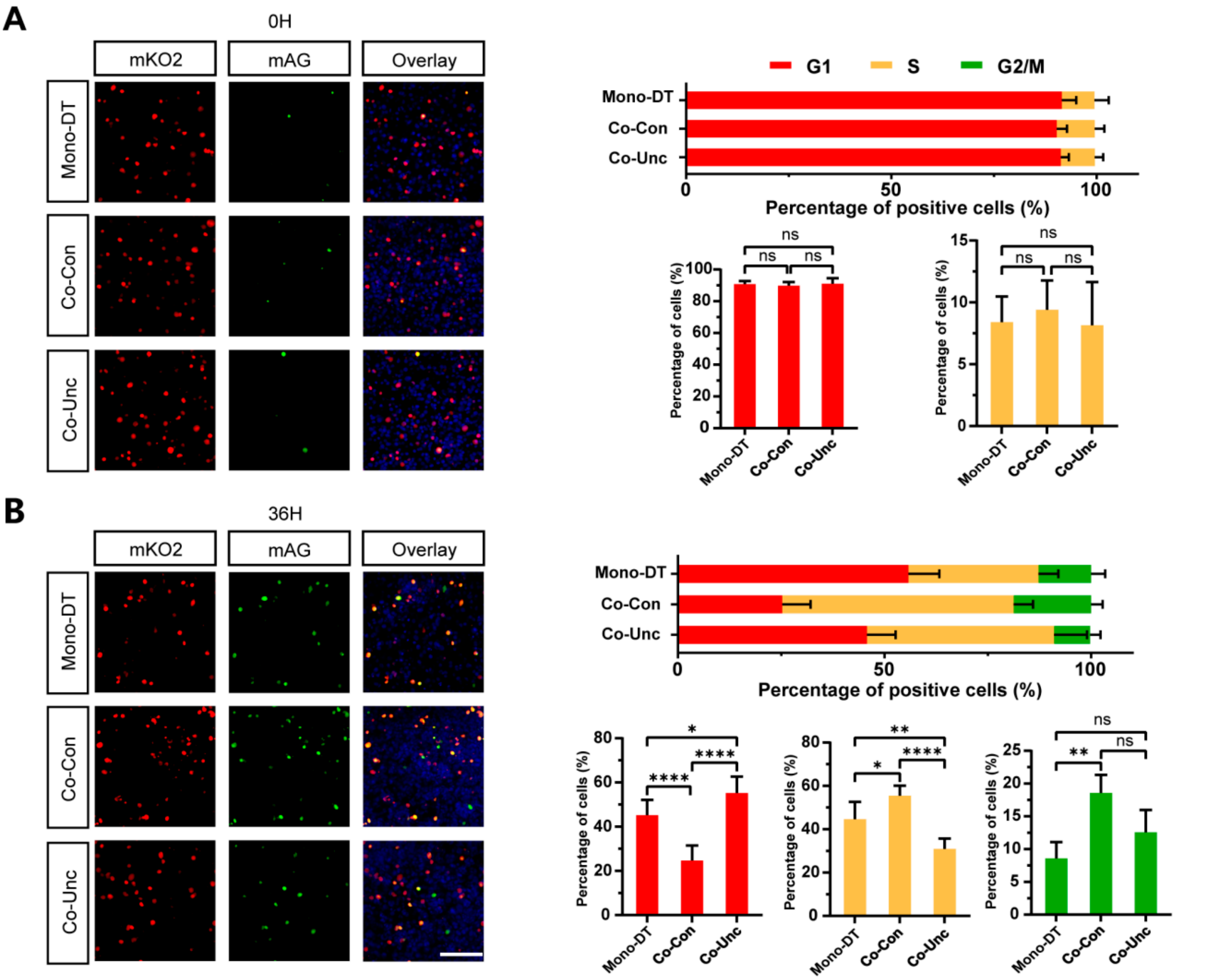

Figure S1. Analysis of cell cycle dynamics of MCF-7 cells in tumor microtissues under different mechanical conditions. (A) FUCCI fluorescence expression of MCF-7 tumor cells in microtissues with different mechanical conditions at 0 h of culture (left) and cell cycle distribution statistics (right). The scale bar is 150 µm. (B) FUCCI fluorescence expression of MCF-7 tumor cells in microtissues with different mechanical conditions at 36 h of culture (left) and cell cycle distribution statistics (right). The scale bar is 150 µm. * $p < 0.05$, ** $p < 0.01$, **** $p < 0.0001$.
